# Epidemiology of Human T-cell lymphotropic viruses (HTLV) in England and Wales, 2003-2012

**DOI:** 10.1186/1742-4690-11-S1-P60

**Published:** 2014-01-07

**Authors:** Sara E Croxford, Valerie C Delpech, Jennifer HC Tosswill, Katy Davison, Graham P Taylor

**Affiliations:** 1 HIV/STI Department, Public Health England (PHE), London, UK; 2 Viral Reference Department (VRD), Microbiology Services, PHE, London, UK; 3 Immunisation, Hepatitis and Blood Safety Department, PHE, London, UK; 4 Imperial College, London, UK; 5 On behalf of the National HTLV Steering Group

## 

We report on the epidemiology of HTLV in England and Wales (E&W) over the past decade. HTLV diagnoses in E&W are reported to PHE by clinicians, laboratories and NHS Blood and Transplant. Confirmatory testing is done by the VRD. Reports received up to the end of January 2013 were included in these analyses. Of the 892 persons diagnosed with HTLV in E&W between 2003-2012, the majority had HTLV-1 (88%; 721/818). Two-thirds were women (64%; 573/889) and 87% were aged ≥35 years (768/887). Where ethnicity was reported (568), black Caribbeans accounted for 59% (337) of diagnoses, followed by whites (21%; 117), black Africans (11%; 66) and persons of Indian-subcontinent ethnicity (3%; 18). 43% (142/338) of people diagnosed probably acquired HTLV infection in the UK and 40% (135/338) in Latin America/ Caribbean. The majority of diagnoses were made among those exposed through mother-to-child transmission and/or heterosexual contact (90%; 381/423). For persons presenting with HTLV-related symptoms (559), the median age was 57 years and 45 years for those without symptoms (331). Median age for persons reported with adult T cell lymphoma (117) was 59 years and 55 years for HTLV-I-associated myelopathy (74). Of 283 symptomatic infections, 87% (247) were among black African/Caribbeans. 75 (8.4%) persons died over the decade, 84% (63) within one year of HTLV diagnosis. Though rare in E&W, HTLV infection is life-long and can result in debilitating disease. Enhanced surveillance remains of public health importance to continue building an epidemiological picture of HTLV and to better understand clinical disease progression.

